# Postoperative Restrictions After Anterior Cervical Discectomy and Fusion

**DOI:** 10.7759/cureus.9532

**Published:** 2020-08-03

**Authors:** Gaetano De Biase, Selby Chen, Mohamad Bydon, Benjamin D Elder, Jamal McClendon, Hugh G Deen, Eric Nottmeier, Kingsley Abode-Iyamah

**Affiliations:** 1 Neurosurgery, Mayo Clinic, Jacksonville, USA; 2 Neurosurgery, Mayo Clinic, Rochester, USA; 3 Neurosurgery, Mayo Clinic, Phoenix, USA

**Keywords:** acdf, cervical brace, activity restrictions, eras, driving

## Abstract

No scientific evidence on restrictions for patients following an anterior cervical discectomy and fusion (ACDF) is available. The goal of this study is to assess the practice and patterns of restrictions after single-level and multilevel ACDF at an academic institution. We submitted two questionnaires, for restrictions after single-level and multilevel ACDF, to 18 spine surgeons at our institution. Questions included length of time in practice, use of cervical collar, postoperative restrictions and practices. We received 10 complete responses. Four (40%) of the respondents were in practice for less than 5 years; 3 (30%) 5 or more years, but less than 10; 1 (10%) 10 or more years, but less than 20; 2 (20%) 20 or more years. Only two (20%) surgeons recommend a cervical collar after a single-level ACDF, while seven (70%) do so after a multilevel ACDF, for an average of 9.1 weeks and standard deviation (SD) of 2.8. Nine surgeons (90%) reported providing lifting restrictions after a single-level and multilevel ACDF, with a mean of 10 kg and SD of 2.5 in both cases. 5 (50%) give driving restrictions after a single-level ACDF, eight (80%) do so after a multilevel. eight (80%) recommend physical therapy after both single-level and multilevel ACDF. three (30%) obtain a CT to confirm fusion at one year. Only two (20%) recommend a bone stimulator. Significant variability exists among surgeons in regards to restrictions following ACDF, but some areas of consensus emerged: 90% of respondents give lifting restrictions, with a mean of 10 kg, 80% recommend physical therapy for a range of motion and muscle strengthening.

## Introduction

Anterior cervical discectomy and fusion (ACDF) is one of the most common procedures performed for the cervical spine. Approximately 132,000 ACDFs are performed annually in the United States [1]. ACDF is indicated for a wide range of pathologies including degenerative disc disease, degenerative spondylosis, radiculopathy, myelopathy, to decompress and fuse with marked improvement in patient outcomes [2-4].

A question frequently asked by patients is when they can go back to their normal life after the procedure (going back to work, driving or what type of activities they should avoid after surgery).

Driving restrictions, lifting and activity limitations can significantly affect the postoperative course of patients that may be unable to go back to work and resume their daily activities. To date, scientific evidence of patients’ ability to safely resume driving after spinal surgery is rather limited and no clear evidence is available in regards to postoperative restrictions for patients that undergo an ACDF; thus surgeon’s recommendations are based on empirical data and personal experience [5].

The purpose of this study was to assess with two questionnaires the restrictions routinely recommended by spine neurosurgeons at a tertiary academic institution after single-level and multilevel ACDF. We hypothesized that given the lack of high-level evidence significant variability could exist.

## Materials and methods

Two identical questionnaires were prepared with Google Forms to record the attitude and preferences of spine neurosurgeons at our institution regarding bracing and restrictions after single-level and multilevel ACDF.

We collected data on how long they had been in practice, followed by questions on whether they use a cervical collar postoperatively and for how long. The following section inquired about postoperative restrictions, whether they give lifting restrictions and how much, whether they give driving restrictions and how long, restrictions on lightweight exercise (e.g., running, yoga, swimming); restrictions on high impact activity (e.g., golfing, weight lifting) and duration of such restrictions; when they use return to work restrictions (office work/light labor/heavy labor) and for how long. We then inquired about whether they prescribed postoperative physical therapy for a range of motion and muscle strengthening; action in case of symptomatic and asymptomatic pseudarthrosis; use of CT confirmation of fusion. We also asked about whether the presence of osteoporosis would change the restrictions and, if so, how; finally, we inquired about the use of a bone growth stimulator and duration of use.

After being preliminarly circulated among the authors to confirm the adequacy of its design, we submitted the questionnaires via email to 18 spine neurosurgeons at our institution.

## Results

Population demographics

**Figure 1:**
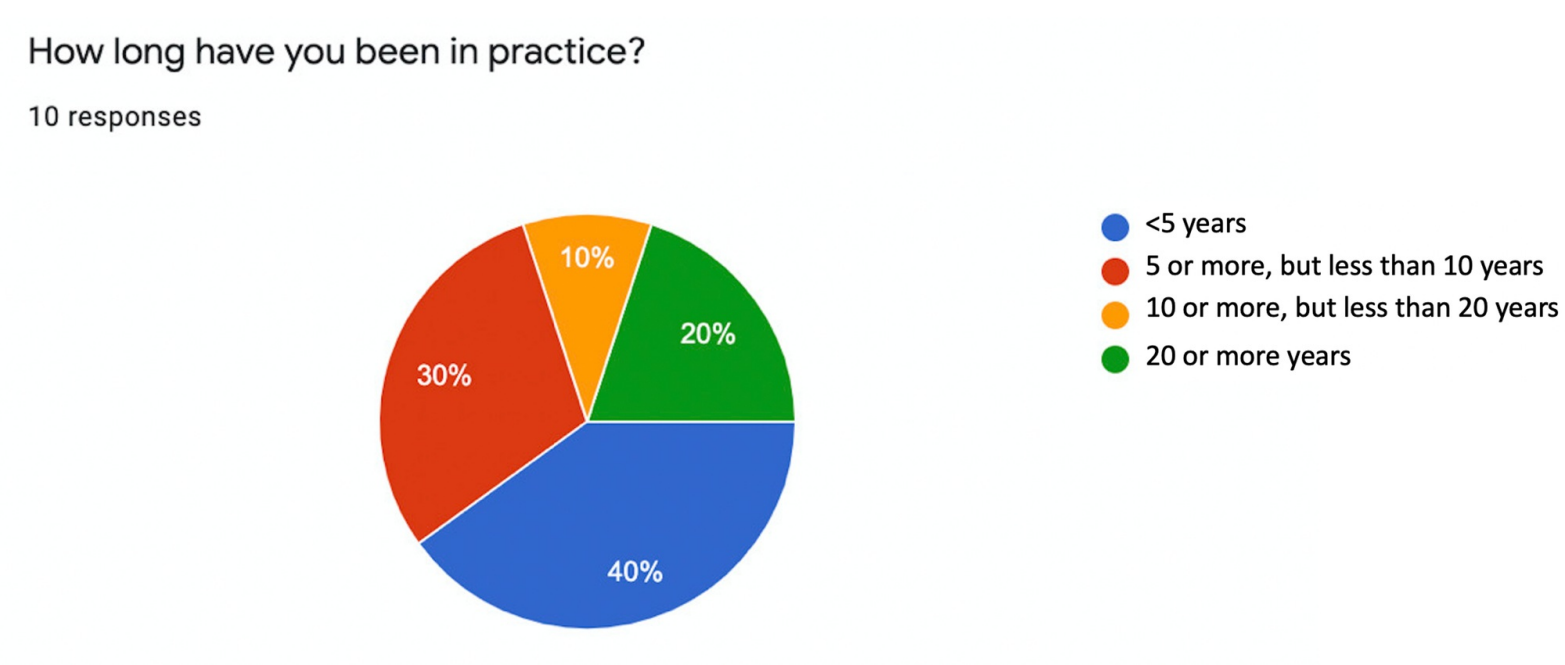
Duration of practice of the questionnaire respondents

We received 10 responses, which corresponds to a response rate of 56%. No surveys had to be excluded due to incomplete information. All the respondents were neurosurgeons working in the United States at a large academic center. Four (40%) of the respondents were in practice for less than 5 years; 3 (30%) 5 or more years, but less than 10 years; 1 (10%) 10 or more years, but less than 20 years; 2 (20%) more than 20 years. In terms of the length of time in practice, according to the biographical information that was collected, 70% of the respondents were in practice for less than 10 years, while 30% were in practice for 10 or more years (Figure 1). 

Bracing frequency

Only two (20%) surgeons routinely recommend a cervical brace after a single-level ACDF, one uses it for two weeks and the other one for eight weeks. Collectively, the postoperative bracing regimen was implemented by seven (70%) respondents after a multilevel ACDF, for an average of 9.1 weeks and standard deviation (SD) of 2.8 weeks, with a range of 6-12 weeks. When comparing surgeons in practice for less than 10 years with those for 10 or more years, the total duration of clinical experience did not appear to influence the propensity of surgeons to brace their patients after ACDFs (71% 0-10 years, 67% 10 or more years).

Postoperative restrictions

**Figure 2:**
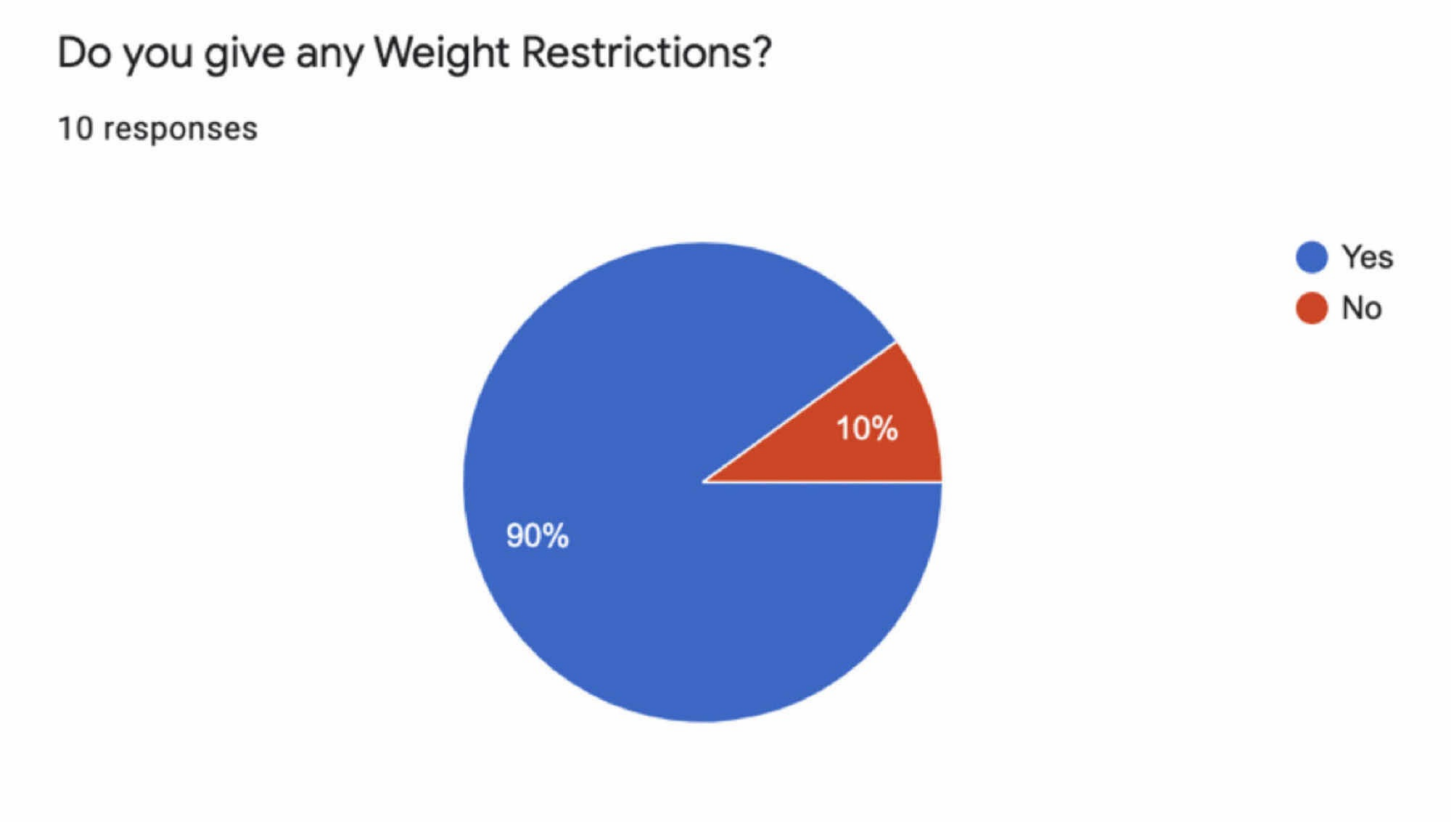
Weight lifting restriction responses after single-level and multilevel ACDF ACDF, anterior cervical discectomy and fusion

Nine (90%) of the respondents give lifting restrictions after a single-level and multilevel ACDF, with a mean of 10 kg and SD of 2.5 kg in both cases, and a range of 5-15 kg (Figure 2). 

Five (50%) give driving restrictions after a single-level ACDF, while eight (80%) do so after a multilevel. The length of the driving restrictions ranges from one to eight weeks for a single-level ACDF, and 4-12 weeks for multilevel ACDFs; two surgeons give restrictions until patients come off narcotics.

Six (60%) give limitations on light weight exercise (e.g. running, yoga, swimming) after a single-level ACDF, for a mean of 10 weeks, SD 2.2 weeks, and range 8-12 weeks; 7 (70%) surgeon do so after a multilevel ACDF, for an average of 13.1 weeks, SD 5 weeks, and range 8-24 weeks.

Nine (90%) recommend restrictions on high impact activities (e.g., golfing, weight lifting) after a single-level and multilevel ACDF. Mean duration after a single level ACDF is 18.7 weeks, SD 15.3 weeks, and range 6-54 weeks; after a multilevel ACDF the average duration is 26 weeks, SD 17.2 weeks, and range 6-54 weeks.

Ten (100%) of the respondents give return to work restrictions in case of heavy labor for both single-level and multilevel ACDF; two (20%) after a multilevel ACDF give return to work restrictions even in case of office work. After single level ACDF, the mean return to work restrictions is 11.3 weeks, SD 5.3 weeks, with a range 6-24 weeks; after a multilevel ACDF, 12.3 weeks, SD 5.7 weeks range 6-24 weeks (Tables 1, 2). 

**Table 1:** Postoperative restrictions after single-level ACDF ACDF, anterior cervical discectomy and fusion

Cervical brace – no. (%)	2 (20)
Lifting restrictions – no. (%)	9 (90)
Weight limit – mean (SD) kg	10 (2.5)
Driving restrictions – no. (%)	5 (50)
Duration – range in weeks	1-8
Restrictions on light exercise – no. (%)	6 (60)
Duration – mean (SD) weeks	10 (2.2)
Restrictions on high impact exercise – no. (%)	9 (90)
Duration – mean (SD) weeks	18.7 (15.3)
Return to work restriction for heavy labor – no. (%)	10 (100)
Duration – mean (SD) weeks	11.3 (5.3)

**Table 2:** Postoperative restrictions after multilevel ACDF ACDF, anterior cervical discectomy and fusion

Cervical brace – no. (%)	7 (70)
Lifting restrictions – no. (%)	9 (90)
Weight limit – mean (SD) kg	10 (2.5)
Driving restrictions – no. (%)	8 (80)
Duration – range in weeks	4-12
Restrictions on light exercise – no. (%)	7 (70)
Duration – mean (SD) weeks	13.1 (5)
Restrictions on high impact exercise – no. (%)	9 (90)
Duration – mean (SD) weeks	26 (17.2)
Return to work restriction for heavy labor – no. (%)	10 (100)
Duration – mean (SD) weeks	12.3 (5.7)

Postoperative practices

Eight (80%) recommend physical therapy (PT) after both single-level and multilevel ACDF. Mean timing for PT is 8.75 weeks since surgery, SD 2.8. Only three (30%) respondents obtain a CT to confirm fusion at one year. In case of asymptomatic pseudarthrosis after single-level and multilevel ACDF, all respondents would simply follow the patients, while in case of symptomatic pseudarthrosis they would either revise or consider a posterior fusion. 4 (40%) in case of osteoporosis would extend the duration of postoperative restrictions. Only two (20%) recommend a bone growth stimulator, one for six months and the other surgeon for nine months.

## Discussion

Postoperative restrictions and limitations are a crucial part of a patient’s recovery following cervical spine surgery. To the best of our knowledge, there are no studies in the literature looking at postoperative restrictions following ACDF. In the absence of scientific data for postoperative lifting restrictions, activity limitations and physical therapy after ACDF surgeon’s recommendations are based on empirical data and personal experience. With the increased prevalence of enhanced recovery after surgery (ERAS) in other disciplines and in neurosurgery (a multidisciplinary, multimodal approach to improving surgical outcomes by using subspecialty- and procedure-specific evidence-based protocols in the care of surgical patients), getting patients back to their normal life and work has become increasingly important [6,7]. To achieve this goal, an assessment of the validity and efficacy of the postoperative restrictions is needed. We, therefore, decided to conduct this questionnaire study to determine the practices and patterns in our academic institution.

Given the lack of clear guidelines, we hypothesized that ample variability would exist among surgeons. Significant consensus was instead found in regards to postoperative lifting restrictions, with 90% of the surgeons giving restrictions both after single-level and multilevel ACDF; seven surgeons recommend no more than 10 kg in both cases, one no more than 5 kg and one no more than 15 kg.

Some studies have looked at Driver Reaction Time (DRT) to try to assess when patients could go back to driving following spine surgery [8,9]. Safely returning to driving has to take into consideration much more than just DRT as postoperative fatigue, cognitive function and sensory-motor coordination also play a critical role. Ample variability was revealed by our questionnaires for driving restrictions. Only 50% of surgeons give driving restrictions after single-level ACDF, ranging from just one week to two months. 80% of the respondents give driving restrictions after multilevel ACDF, with a wide range of duration from four weeks to three months. Interestingly, two surgeons give driving restrictions only until patients come off narcotics.

As far as limitations on high impact activities (e.g., golfing, weight lifting) after a multilevel ACDF, 90% of the respondents agreed on giving restrictions, but significant variability was observed in the duration, as shown by the range of 6-54 weeks and SD of 17.2 weeks. All the respondents give return to work restrictions in case of heavy labor for both single-level and multilevel ACDF: common practice seems to be 8-12 weeks.

The lack of consensus among surgeons regarding the efficacy of postoperative bracing is reflected in our study by the fact that only 20% of the respondents use a cervical brace after a single-level ACDF and 70% after a multilevel ACDF [10,11]. Four (40%) of surgeons extend the duration of postoperative restrictions in osteoporotic patients; smoking was also reported as a factor affecting the length of restriction and use of cervical bracing. Only a minority of surgeons say they routinely use a CT scan at one year to assess fusion and only two respondents recommend a bone growth stimulator.

Carragee et al. conducted a small prospective clinical trial to assess the necessity of postoperative activity restrictions after posterior lumbar discectomy and found that lifting of postoperative restrictions after limited discectomy allowed shortened sick leave without increased complications [12]. Further characterization of the efficacy and necessity of restrictions after single-level and multilevel ACDF is in our opinion warranted to establish the most appropriate practices.

We acknowledge that there are several limitations to this study. Because the questionnaires required participants to assess their own practice patterns, their responses may be subject to a recall bias, which may affect the accuracy of the results.

The aim of this study was to analyze the postoperative activity restriction patterns at our academic institution, and give the sample size and characteristics we do not state that this represents the opinion and preferences of the neurosurgical spine community at large. By surveying a more diverse cohort of surgeons, fellowship and non-fellowship trained, academic and private practice, from the United States and international, it will be possible in future studies to establish more definitive conclusions regarding the issue of postoperative restrictions.

Our study revealed, even among academic neurosurgeons working at the same institution, significant variability in postoperative restrictions following ACDF. A potential explanation for the differences among surgeons from the same institution could be the variability occurring from different training programs of origin. Some areas of consensus emerged, including lifting restrictions, with 70% of the surgeons recommending no more than 10 kg after single-level and multilevel ACDF, and return to work recommendations in case of heavy labor, for which common practice seems to be 8-12 weeks.

## Conclusions

To date, scientific evidence of what limitations should be followed after an ACDF is rather limited. Our questionnaire study revealed significant variability among surgeons in restrictions following single-level and multilevel ACDF. We found consensus in lifting restrictions, with 90% of surgeons giving lifting restrictions, with a mean of 10 kg, and return to work recommendations in case of heavy labor for which common practice seems to be 8-12 weeks. Additional characterization of the efficacy and necessity of restrictions after single-level and multilevel ACDF is, in our opinion, warranted.
